# Notes and News

**Published:** 1889-07-27

**Authors:** 


### Notes and News.

Collected and Communicated.

The Hospital Sunday Fund is over £39,000, and is likely to exceed last year's by a considerable amount. It is whispered that the increase is partly due to a West End freak, several ladies and gentlemen belonging to the fashionable world having devoted their evenings to wandering about the streets as a guitar band, giving the proceeds of their performances to the Hospital Sunday Fund.

It was at the following places that the ladies engaged in the Hospital Saturday street collection realised the largest sums:—Capel-court, £73; Charing-cross Railway Station, £50; Finch-lane, £28; Cannon-street Railway Station, £24; St. Swithin's-lane, £21; Liverpool-street Railway Station, £18; Guildhall Tavern, £16; Fenchurch-street Railway Station, £15; Streatham Railway Station, £15.

The "After-Care" Association, which takes care of poor and friendless female convalescents on leaving asylums for the insane, does a great deal of good in so unpretending a way that it does not receive the support it deserves. A little thought will make it obvious that many people, though discharged from an asylum as sane, are not quite fit to take up their ordinary life and work. Their nerves are still unstrung, and the mere necessity of fighting the battle of life without some gradual preparation for the warfare may send them back into their old condition; while a few weeks spent in some pleasant half-way house may restore them entirely to health and strength. The association, which has so treated about fifty patients during last year, has the sympathy of many well-known men, who devote their attention to insanity; it is to be regretted that its work and merits are not better known to the public. The Rev. Henry Hawkins, chaplain of Colney Hatch, is the honorary secretary; the active duties of the post are fulfilled by Mr. H. Thornhill Roxby, and the Earl of Meath is president.

We have two distinguished guests just now in England—Dr. Tholozan, the Shah's physician, and Dr. Nichols, of New York. Dr. Nichols has come over to see our lunatic asylums, especially Edinburgh, as he is to be head of the large asylum to be shortly built at Bloomingdale in the States. It is rather rough on Dr. Tholozan that his name should be similar to that of the hero of "The Fatal Phryne," who, in revenge, turns one of his patients blue by a long course of mercury.

The Committee of the Royal Masonic Institution for Boys at Wood Green have made a full investigation of the charges made against the management, and put all once more on a perfectly open and satisfactory footing. May we remind the managers of the orphan school at Ham that all charities ought to be equally ready to enquire into all charges, and that the resignation of Lady Ellis and the ladies of the committee is a serious step. St. John's Hospital scandal is a proof of how necessary it is to investigate charges immediately they are formulated.

A fever hospital has just been completed and opened at Lynn; it cost £330, and is very small. The ward is 32ft. by 22ft. and 15ft. high, and contains 12,000 cubic feet of air space. The walls and ceiling are plastered, and the floor is of concrete. When anyone suffering from a dangerous contagious disease arrives on board ship in the roads, the medical officer of health goes down and makes an examination, and the patient is brought to Lynn, taken up the river Nar in a boat, and from thence on a stretcher to the hospital. At the west end of the ward in the new building is a double door, so that the patient can be carried quite into the room. Every precaution has been taken to secure perfect ventilation, and under each of the six beds is an inlet for fresh air.

August 5 will be Bank Holiday, and the Seaside Camp for London Boys hopes to entertain some 500 guests that day, if it can raise funds to pay their fares down. The camp is on the Sandhills, close to Deal, and many of our white-faced London lads between the ages of fourteen and seventeen are spending their holidays in its healthy atmosphere this year. If anything can do the stunted moral and physical nature of an ordinary London boy good it would be a fortnight by the sea, with plenty of outdoor exercise and the gentle discipline of "playing at soldiers" which the camp offers. Let us hope that those luckless youths whose sole holiday will be from Saturday to Tuesday the first week in August will at least be able to have two days at the camp.

The Prince of Wales laid the foundation-stone of the new Samaritan Free Hospital in Marylebone-road on Wednesday. The new building is not far from the old one, and is to cost £12,000. The Prince was received with cheers, and the band of the 5th Middlesex Volunteers played the National Anthem. The Prince was received by Dr. H. Savage, founder of the hospital, Dr. Routh, Sir Spencer Wells, Dr. Day, Dr. Percy Boulton, Dr. Granville Bantoch, and others. Miss Susan Grosvenor, the pretty little granddaughter of Lord Ebury, presented the Princess of Wales with a handsome bouquet of orchids. In the absence of Lord Leigh, through a death in the family, the Earl of Selborne read an address begging the Prince to lay the memorial-stone. For forty-two years the hospital has done good work, and it was the surgeons of the Samaritan who demonstrated to the world that the operation of ovariotomy could completely restore certain poor women to health. The Prince made one of his usual neat little speeches. The Princess received purses from Lady Laurence, Miss Butler, of Lower Seymour-street, Miss Tidy, of Dorset House, and others. After a short service, presided over by the Bishop of Marlborough, the audience dispersed.

The following vacancies are announced this week, the amount of the salary in each case being given after the name of the institution :

**Resident Medical Officers.**

Manchester Consumption Hospital.—Medical Officer.—£60, board, &c.
Deaconesses' Institution, Tottenham. House Surgeon.—£25, board, &c.
Royal London Ophthalmic Hospital. Junior House Surgeon. £50, board, &c.
Nottingham Borough Asylum. Locum Tenens for August.
Shadwell Children's Hospital. House Surgeon.
Hospital for Women, Soho-square. House Physician.—£75, board, &c.

**Non-Resident Medical Officers.**

City of London Hospital, Victoria Park. Assistant Physician.
Bradford Medical Aid Association. Assistant.—£120.
Nottingham. Medical Officer of Health.
Royal College of Physicians. Milroy Lecturer.
Leeds Friendly Societies. Surgeon.—£200, residence, &c.

---

An old woman named Mary Bamber, living at Adlington, near Chorley, died last week from hydrophobia. Two months ago she was bitten on the arm by a cat; the wound healed, but symptoms of hydrophobia developed and she died in terrible agony. Mr. Farquharson made this and other cases the reason for enlivening the House of Commons by asking whether cats should not be included in the muzzling order. Mr. Matthews replied that as cats used their claws as well as their teeth he feared the muzzling order would be useless even if possible to be carried out.

---

Sir James Paget lately opened the Eye Hospital at Oxford, which owes its existence chiefly to the efforts of Dr. Doyne. In the course of his speech, Sir James Paget said he could not and would not discuss the question of special hospitals in general (rather Irish !), but that of the necessity of the Oxford Eye Hospital he had no doubt. Years and years ago, when he first began surgical practice, he took his fair share of the treatment of the eye, but before eight or ten years had passed he found that unless he could give to it far more time and attention than he could afford from other subjects that came under his care, it was impossible that he should nearly approach to those who were giving all the attention it deserved—(applause)—so gradually he retired from that branch ; and from that time to this he had never, in the ordinary expression, "seen an eye," and he should think there were few persons more ignorant than himself at that time as to what should be done for an eye seriously injured or diseased.

---

Prince Albert Victor had a hearty reception at Harrogate when he opened the Bath Hospital and Convalescent Home. The town was gorgeously decorated and full of visitors, and the weather was all that could be desired. The Prince had not only to make a speech at each building, but he had to return thanks for the Royal Family at a dinner in the evening. The hospital is a pretty building. Both outside and inside a cheerful domestic appearance has been aimed at. The great difference in the levels of the land (upwards of twenty feet), which contributes much to the picturesqueness, occasioned many difficulties in carrying out the original design. The graduated terraces and balcony on the south side form delightful airing places for inmates who may be unable to take exercise in the ornamental gardens and grounds, or tennis courts ; and the winter garden will afford light and recreation during damp or inclement seasons. There is ample accommodation for 150 inmates. The convalescent home is so planned that it can be readily extended, if necessary, at a future time. The building has been carried out from the designs and under the superintendence of the architects, Messrs. Worthington and Elgood, of Manchester and London.

Through the kindness of a lady who sent £5 anonymously to the matron, Miss Foster, a party of 19 in-patients and nurses from the Hospital for Epilepsy and Paralysis, Regent's Park, recently spent a long day at Herne Bay. There are, doubtless, within the walls of London hospitals, many other patients, more or less convalescent, who would gladly be drafted off for similar expeditions, and many other matrons who would gladly have the same means for organising them, were the necessary funds sent to the hospitals.

---

We wish someone would tell us what the trustees of the Working Women's Benefit Society at Oxford do ; we know quite well what they say. Never is any scheme for the good of the gentler sex started but these same trustees write to the papers to protest or demur. Just now they protest against the United Sisters' Friendly Society because it is not exactly on their lines and their advice was never asked. Raising stumbling-blocks and protesting in newspapers is not likely to be of practical benefit to women. Truly many must say of this society, "Methinks they do protest too much."

---

Princess Christian, whose charity is ever graciously bestowed, was present last week at an entertainment in Brompton Hospital, organised for the delectation of the patients, and manifested her interest by contributing two pianoforte solos and by taking part in a duet with Miss Mary Liddell. The Ladies' Guitar Band, which numbered among its members Lady Dorothea Murray and Ladies Emily and Sophie Cadogan, discoursed sweet music with effect; Lady Feodorowna Sturt sang two songs, and the Hon. Winifred Sturt played two violin solos ; while the Hon. A. Yorke gave recitations.

---

The ways of the Post Office are peculiar. A correspondent sent us a copy of the *Eastern Daily Press* of the 15th inst., containing a report of the quarterly meeting of the Governors of the Norfolk and Norwich Hospital. The concluding paragraph, which reports a discussion resulting in power being given to the house-surgeon to supersede the lady superintendent in nursing matters should a nurse be guilty of gross negligence of duty, insolence, or insubordination, was embellished with the word "enlightened," and four notes of exclamation at the end, in red ink. For this act on the part of our correspondent, the Post Office authorities charged us threepence, on the ground that the newspaper contained a communication of the nature of a letter. The young men at St. Martins-le-Grand must be a little hard up for something to do just now we imagine.

---

Charity concerts are but mournful entertainments ; seats half empty, and half the entertainers to be apologised for. It is a pity those who buy the seats do not give them away if they cannot use them ; it is a pity the eminent artistes who promise to help do not keep their promises. To turn from the general to the particular. The Central London Ophthalmic offered an attractive programme at Steinway Hall on the 17th, and Miss Eleanor Rees fulfilled her engagement and sang with great skill and taste. Mrs. Frank Snoad had written a few pathetic verses for the occasion, entitled "No Room," and some clever friend had designed an amusing frontispiece for the programme. On the 18th, there was a concert at the National Hospital in Queen-square which wishes to be rid of its chapel debt. The hospital was looking its very best, and the chapel was beautifully decorated and sweet with the scent of large white lilies. Mdlle. Sylvia de Martini gave some quaint little guitar songs, and Mr. Arthur Thompson sang well. The nurses sold the programmes and showed the visitors round the wards.

